# Detection of nerve agent stimulants based on photoluminescent porous silicon interferometer

**DOI:** 10.1186/1556-276X-7-527

**Published:** 2012-09-25

**Authors:** Seongwoong Kim, Bomin Cho, Honglae Sohn

**Affiliations:** 1 Department of Chemistry, Chosun University, 375 Seosuk-dong, Dong-gu, Gwangju 501-759, South Korea

**Keywords:** Photoluminescence, Porous silicon, Fabry-Pérot, Organophosphate, Sensor

## Abstract

Porous silicon (PSi) exhibiting dual optical properties, both Fabry-Pérot fringe and photolumincence, was developed and used as chemical sensors. PSi samples were prepared by an electrochemical etch of p-type silicon under the illumination of 300-W tungsten lamp during the etch process. The surface of PSi was characterized by cold field-emission scanning electron microscope. PSi samples exhibited a strong visible orange photoluminescence at 610 nm with an excitation wavelength of 460 nm as well as Fabry-Pérot fringe with a tungsten light source. Both reflectivity and photoluminescence were simultaneously measured under the exposure of organophosphate vapors. An increase of optical thickness and quenching photoluminescences under the exposure of various organophosphate vapors were observed.

## Background

Since the discovery of visible photoluminescence from nanocrystalline porous silicon (PSi) [1], PSi has been intensively investigated for a variety of applications such as chemical [2] and biological sensors [3] and drug delivery system [4], especially that PSi is an ideal candidate for gas- or liquid-sensing applications since it has a very large specific surface area on the order of few hundreds of square meters per cubic centimeter. The main techniques investigated to achieve signal transduction are capacitance [5], resistance [6], photoluminescence [7], and reflectivity [8]. Typically, PSi prepared from p-type silicon wafer under dark condition exhibits well-defined Fabry-Pérot fringes in the optical reflectivity spectrum. However, luminescent PSi is usually prepared by the photoetch of n-type silicon wafer. Condensation of organic vapors in the pores can lead to a shift in the Fabry-Pérot fringes by modification of the refractive index of PSi films [9]. This property has been exploited to develop PSi sensors for the detection of toxic gases [10,11], solvents [12], DNA [13], and proteins [3,14,15]. Organic vapors have been detected quantitatively by quenching of photoluminescence of the quantum-confined Si crystallites in PSi [16]. The detection of chemical warfare agents is of major importance since they are highly toxic and, thus, a matter of international concern. The LCt_50_ for sarin by inhalation of the vapor form is 100 mg of sarin/m³ of air for 1 min. TEP, DMMP, and DEEP are stimulants for G-type nerve agents. For the detection of toxic gases, a specific as well as a sensitive and rapid detection is of current interest. Recently developed methods and materials for the detection of nerve agents are based on enzymes [17], interferometry [2], fluorescence [18], single-walled carbon nanotube [19], organic polymers [20], and quantum dots [21]. PSi is also an alternative candidate to detect chemical nerve agents. Here, we prepared PSi samples exhibiting both strong photoluminescence and well-defined Fabry-Pérot fringes. Both photoluminescence and reflectivity were measured for the detection of nerve agent stimulants in the gas phase.

## Methods

### Preparation and treatment of PSi

Boron-doped p-type silicon wafers (B-doped, orientation <100>, Siltronix, Inc., Archamps, France) with a resistivity in the range of 1 to approximately 10 Ω·cm were used to fabricate photoluminescent PSi by an anodic etch in ethanolic HF consisting of a 1:1 volume mixture of aqueous 48% hydrofluoric acid (Sigma-Aldrich Corporation, St. Louis, MO, USA) and absolute ethanol (Sigma-Aldrich). The galvanostatic etch was carried out in a Teflon cell using a two-electrode configuration with a Pt counter electrode. PSi was prepared at an anodization current of 100 mA/cm^-^² for 3 min. The anodization current was supplied by a Keithley 2420 high-precision constant current source (Keithley Instruments Inc., Cleveland, OH, USA). Galvanostatic etching was performed under the illumination with a 300-W tungsten filament bulb for the duration of etch. All samples were then rinsed several times with ethanol and dried under argon atmosphere prior to use. The samples were then mounted in a glass chamber connected to a Schlenk line. The Schlenk line was connected to a direct-drive vacuum pump. The chamber was pumped to <1 mTorr between gas exposures.

## Materials

For analyte exposure studies, dimethyl methylphosphonate (DMMP, 97%, Sigma-Aldrich), diethyl ethylphosphonate (DEEP, 98%, Sigma-Aldrich), and triethyl phosphate (TEP, 99.8%, Sigma-Aldrich) were purchased and used without posttreatment. Stock solutions of the molecules were prepared by freeze-pump-thaw degassed three times prior to use. Cu(II)-tetramethylethylenediamine (TMEDA) complex was obtained from the reaction of cupric sulfate (99%, Sigma-Aldrich) and TMEDA (99%, Sigma-Aldrich) in methanol. PSi samples were spin-coated with 0.01 M of copper (II)-TMEDA aqueous solution, washed with acetone to remove the copper complex on the surface of PSi, and dried under reduced pressure prior to use.

### Photoluminescence and reflectance measurements

Steady-state photoluminescence spectra were obtained with an Ocean Optics S2000 spectrometer (Ocean Optics, Inc., Dunedin, FL, USA) fitted with a fiber optic probe. The excitation source was a UV LED (*λ*_max_ = 460 nm) focused on the sample (at a 45° angle to the surface normal) by means of a separate fiber. Light was collected at a 90° angle to the incident light source with a fiber optic. Spectra were recorded with a CCD-detector in the wavelength range of 400 to 900 nm. Values of percent quenching are reported as *(I*_0_ *− I)/I*_0_, where *I*_0_ is the intensity of the luminescence of PSi, integrated between 400 and 900 nm, in the absence of quencher, and *I* is the integrated intensity of luminescence of PSi in the presence of a quencher. Interferometric reflectance spectra of PSi samples were recorded using an Ocean Optics S2000 spectrometer. A tungsten light source was focused onto the center of a PSi surface. Spectra were recorded with a CCD detector in the wavelength range of 400 to approximately 1,200 nm. The illumination of the surface as well as the detection of the reflected light was performed along an axis coincident with the surface normal. At least three times of measurements were performed for each analyte studied. The morphology of PSi was observed with FE-SEM (S-4700, Hitachi, Ltd., Chiyoda, Tokyo, Japan).

## Results and discussion

**Figure 1:**
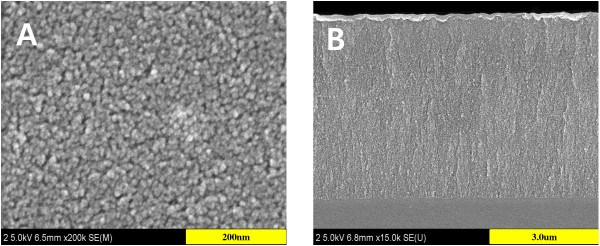
Surface (A) and cross-sectional (B) SEM images of photoluminescent PSi.

An anodic etch of p-type silicon wafer with resistivities of 1 to approximately 10 Ω·cm in ethanolic HF solution generally produces PSi single layer with a network of microporous rather than mesoporous or macroporous and exhibits Fabry-Pérot fringe pattern in the optical reflectivity spectrum. The pore size of p-type PSi can be increased by increasing the concentration of the dopant and decreasing the aqueous HF concentration. In this work, PSi exhibiting dual optical properties, both Fabry-Pérot fringe (optical reflectivity) and photoluminescence, has been successfully fabricated by a galvanostatic electrochemical etch of p-type silicon wafer under the illumination with a 300-W tungsten filament bulb for the duration of etch. The surface and cross-sectional morphologies of PSi are obtained with cold FE-SEM and shown in Figure 1. FE-SEM image of the PSi surface indicated that the PSi prepared under tungsten-halogen white light illumination exhibited a very stable and even surface. FE-SEM image of PSi indicates that the prepared PSi has cylindrical mesopores with a pore size of few nanometers and a depth of few microns.

**Figure 2:**
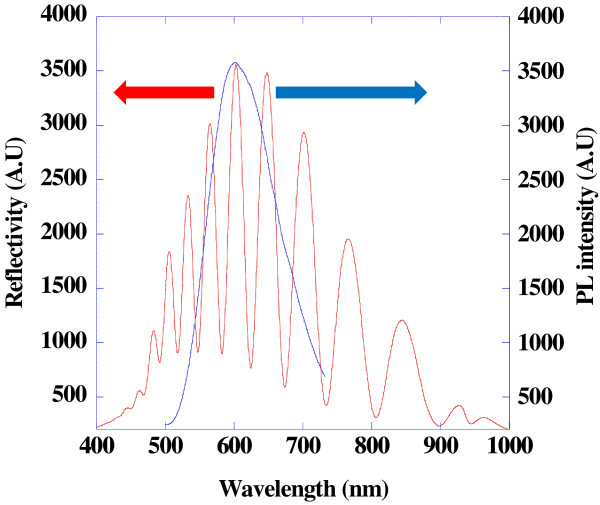
Reflectivity (red line) and photoluminescence (blue line) spectra of photoluminescent PSi.

PSi consisted of a large number of interconnected Si nanocrystallites with a surface that is almost entirely covered with hydrogen atoms. Optical reflectance and photoluminescence spectroscopy were used to investigate the optical properties of PSi. Prepared PSi displayed well-resolved Fabry-Pérot fringes in reflectometric interference spectrum. Photoluminescence spectra were measured and collected at room temperature as shown in Figure 2. PSi samples exhibited a strong visible orange photoluminescence possibly due to the photoetch. The maximum intensity of emission spectrum was centered at 610 nm with an excitation wavelength of 460 nm. The photoluminescence is due to the quantum confinement of silicon nanocrystallites in PSi.

The optical spectrum from PSi is governed by the Fabry-Pérot relationship. The wavelength of a peak in the reflectivity spectrum is given by Equation 1:

(1) mλ=2nL

where *m* is the spectral order of the optical fringe; *λ*, the wavelength; *n*, the refractive index of the film; and *L*, its thickness. The product *nL* is the quantity referred to as ‘optical thickness’ (OT). Any change in the refractive index *n* will induce a proportional shift of the position of the interference fringe position according to Equation 2:

(2) mΔλ=2ΔnL

The value of *nL* from Equation 2, OT, is obtained from the position of the peak in the Fourier-transformed plot of reflected intensity versus frequency. The detailed method to obtain OT using FT from the WaveMetrics (Lake Oswego, OR, USA) was reported by Sailor et al. [22]. Since the parameter for PSi is fixed, change in optical thickness (*Δ*OT) depended on the refractive index and vapor pressure of an individual analyte as well as molecular polarity. When a PSi interferometer is exposed to analytes in the gas phase, adsorption or capillary condensation induces an increase of its effective refractive index by replacement of a fraction of air (*n* = 1) by a fraction of analyte (*n* > 1).

**Figure 3:**
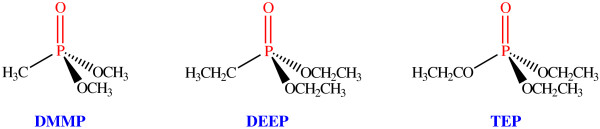
Chemical structures of nerve agent stimulants.

**Figure 4:**
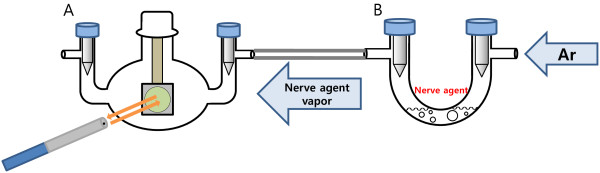
**Schematic diagram for the detection of nerve agent simulants.** (**A**) Nerve agent vapor generator and (**B**) detection chamber.

**Figure 5:**
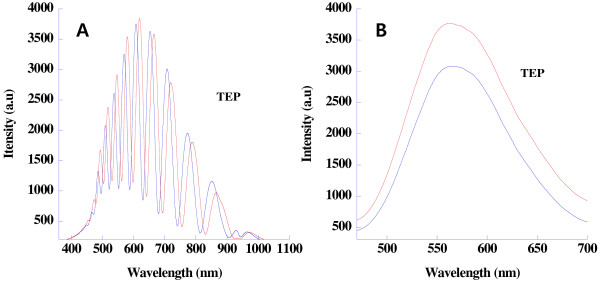
**Shift of Fabry-Pérot fringe pattern (A) and quenching photoluminescence spectra (B).** Under the exposure of TEP vapors.

Figure 3 shows the chemical structures of TEP, DMMP, and DEEP, respectively. Schematic diagram for the detection of nerve agent stimulants is shown in Figure 4. The PSi sample is exposed to a flux of nerve agent stimulants such as DMMP (partial pressure of 0.22 Torr or 290 ppm), TEP (135 ppm), and DEEP (131 ppm) vapor in air with a flow rate of 1 L/min. When the PSi sample is exposed to analytes in the gas phase, capillary condensation causes an increase of its effective refractive index by the replacement of air to organophosphate analyte. Figure 5 shows the change of Fabry-Pérot fringe pattern of PSi while exposing to TEP vapor. A reversible shift of Fabry-Pérot fringe to the longer wavelength under the exposure of TEP vapor is observed. The analytes have different refractive indices and vapor pressures, respectively. The amplitude of the redshift of the reflection peaks depends on both the refractive index and vapor pressure of the analyte condensed in PSi sample as well as the surface characteristics and varied pore diameter.

The change in photoluminescence is measured under the exposure of vapors of nerve agent stimulants. Exposure of PSi to TEP vapors resulted in the quenching of photoluminescence from the luminescent chromophore in PSi to a weakly chemisorbed organophosphate molecule. The intensity of photoluminescence depended on the presence of surface adsorbates. Meyer and Ko observed reversible dynamic quenching of PSi photoluminescence by organic molecules [23].

Cu (II) complex was found to adsorb NH_3_, SO_2_, and organophosphate. PSi sample spin-coated with 0.01 M of Cu-TMEDA aqueous solution was served for the detection of organophosphates. An irreversible shift for the Fabry-Pérot fringe and quenching photoluminescence under the exposure of TEP vapor is observed. However, the little change in the OT and photoluminescence is observed for the PSi with Cu complex. This might be due to the hydrophobic property of Cu complex. Test sets for different organophosphate analytes were analyzed by both quenching photoluminescence and *Δ*OT. The results for the change in OT and quenching photoluminescence under the exposure of different organophosphate analytes were summarized in Table 1.

**Table 1:** Change in OT and quenching photoluminescence under the exposure of different organophosphate analytes

**Organic compounds**	**Concentration (10**^**−6**^ **g/mL)**	***Δ*OT (nm)**			**Quenching photoluminescence (%)**		
		**Entry 1**	**Entry 2**	**Entry 3**	**Entry 1**	**Entry 2**	**Entry 3**
DMMP	290	60	62	62	14.34	14.56	14.31
DEEP	131	123	121	120	13.04	13.08	13.81
TEP	135	205	200	204	14.78	14.38	15.13
DMMP with Cu(II) complex	290	15	16	16	9.19	9.7	9.31
DEEP with Cu(II) complex	131	31	31	29	8.12	8.71	8.61
TEP with Cu(II) complex	135	126	121	123	12.6	12.11	12.8

**Figure 6:**
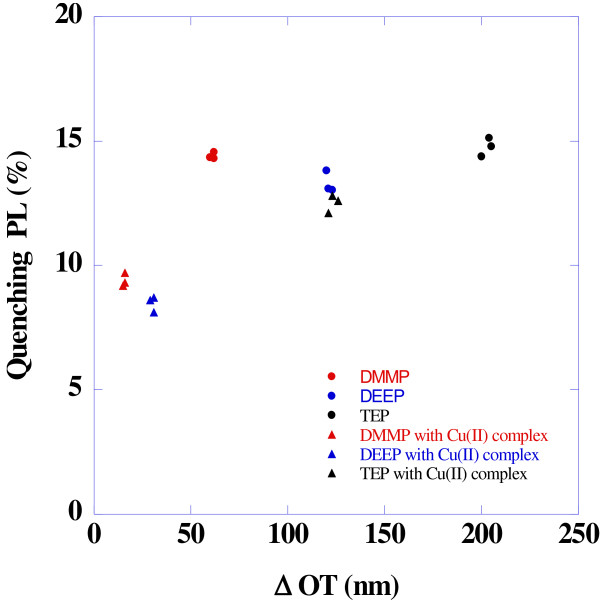
**Plot for the relationship between *Δ*OT and quenching photoluminescence (%).**

Plot for the relationship between *Δ*OT and quenching photoluminescence (%) is shown in Figure 6. It should be noted that TEP shows a larger redshift even if its concentration is lower than DMMP, due to the complex nature of parameters. However, the photoluminescence quenching varied very little, even though the vapor pressure of analytes is different.

**Figure 7:**
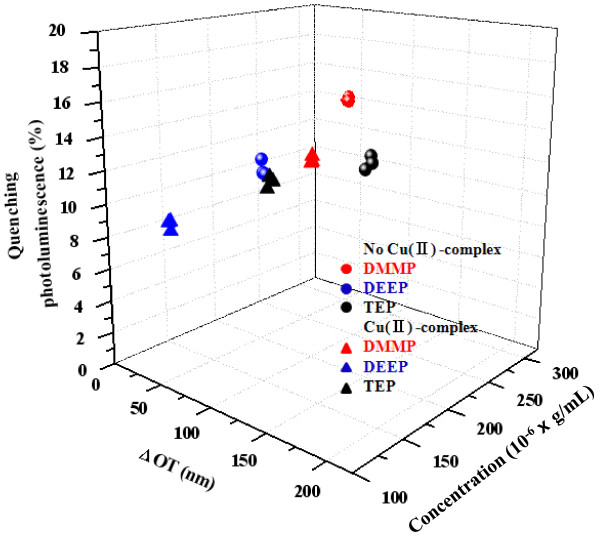
**3D plot.** Showing the relationship between the *Δ*OT, *Δ*PL, and the concentration of analytes (DMMP, 290 × 10^−6^ g/mL; DEEP, 131 × 10^−6^ g/mL; and TEP, 135 × 10^−6^ g/mL) in photoluminescent PSi with or without Cu complex under exposure to three different analytes.

Figure 7 showed the three-dimensional relationship between the *Δ*OT, quenching photoluminescence (*Δ*PL), and concentration of analyte in photoluminescent PSi with or without Cu complex under exposure to three different analytes. PSi sample was exposed to each analyte three times. Each analyte was represented by a cluster of points. The smaller the area of the clusters indicated the better the reproducibility, and the larger the distance between different clusters indicated the better the discrimination. These results demonstrated the potentiality of PSi sensors for the detection of nerve agent simulant.

## Conclusions

PSi displaying both strong orange photoluminescence in the visible region and well-defined Fabry-Pérot interferometric fringes in the optical reflectivity spectrum were fabricated by an electrochemical etch under the illumination for the duration of etch and used for the detection of organophosphate vapors. PSi samples exhibited a strong visible orange photoluminescence at 610 nm with an excitation wavelength of 460 nm. A reversible shift of Fabry-Pérot fringe to the longer wavelength and quenching photoluminescence for the detection of organophosphate vapor is observed. PSi incorporated with Cu-TMEDA complex displayed an irreversible shift for the Fabry-Pérot fringe and quenching photoluminescence. The three-dimensional relationship between the change in optical thickness, quenching photoluminescence, and vapor pressure of analyte displayed a cluster of points which demonstrated the potentiality of PSi sensors for the detection of nerve agent simulant.

## Competing interests

The authors declare that they have no competing interests.

## Authors' contributions

The experiments presented in this work were designed by HS. The PSi was fabricated and characterized optically by SK and BC. HS wrote the manuscript. All authors read and approved the final manuscript.
